# Inappropriate use of antibiotics for childhood diarrhea case management — Kenya, 2009–2016

**DOI:** 10.1186/s12889-019-6771-8

**Published:** 2019-05-10

**Authors:** Chulwoo Rhee, George Aol, Alice Ouma, Allan Audi, Shadrack Muema, Joshua Auko, Richard Omore, George Odongo, Ryan E. Wiegand, Joel M. Montgomery, Marc-Alain Widdowson, Ciara E. O’Reilly, Godfrey Bigogo, Jennifer R. Verani

**Affiliations:** 1 Division of Global Health Protection, Center for Global Health, Centers for Disease Control and Prevention, Atlanta, GA USA; 2 Center for Global Health Research, Kenya Medical Research Institute, Kisumu, Kenya; 3 Center for Global Health Research, Kenya Medical Research Institute, Nairobi, Kenya; 4 Division of Global Health Protection, Centers for Disease Control and Prevention, Nairobi, Kenya; 5 Division of Foodborne, Waterborne, and Environmental Diseases, National Center for Emerging and Zoonotic Infectious Disease, Centers for Disease Control and Prevention, Atlanta, GA USA

**Keywords:** Diarrhea, Guideline adherence, Antibiotics, Dysentery

## Abstract

**Background:**

Antibiotics are essential to treat for many childhood bacterial infections; however inappropriate antibiotic use contributes to antimicrobial resistance. For childhood diarrhea, empiric antibiotic use is recommended for dysentery (bloody diarrhea) for which first-line therapy is ciprofloxacin. We assessed inappropriate antibiotic prescription for childhood diarrhea in two primary healthcare facilities in Kenya.

**Methods:**

We analyzed data from the Kenya Population Based Infectious Disease Surveillance system in Asembo (rural, malaria-endemic) and Kibera (urban slum, non-malaria-endemic). We examined records of children aged 2–59 months with diarrhea (≥3 loose stools in 24 h) presenting for care from August 21, 2009 to May 3, 2016, excluding visits with non-diarrheal indications for antibiotics. We examined the frequency of antibiotic over-prescription (antibiotic prescription for non-dysentery), under-prescription (no antibiotic prescription for dysentery), and inappropriate antibiotic selection (non-recommended antibiotic). We examined factors associated with over-prescription and under-prescription using multivariate logistic regression with generalized estimating equations.

**Results:**

Of 2808 clinic visits with diarrhea in Asembo, 2685 (95.6%) were non-dysentery visits and antibiotic over-prescription occurred in 52.5%. Of 4697 clinic visits with diarrhea in Kibera, 4518 (96.2%) were non-dysentery and antibiotic over-prescription occurred in 20.0%. Antibiotic under-prescription was noted in 26.8 and 73.7% of dysentery cases in Asembo and Kibera, respectively. Ciprofloxacin was used for 11% of dysentery visits in Asembo and 0% in Kibera. Factors associated with over- and under-prescription varied by site. In Asembo a discharge diagnosis of gastroenteritis was associated with over-prescription (adjusted odds ratio [aOR]:8.23, 95% confidence interval [95%CI]: 3.68–18.4), while malaria diagnosis was negatively associated with antibiotic over-prescription (aOR 0.37, 95%CI: 0.25–0.54) but positively associated with antibiotic under-prescription (aOR: 1.82, 95%CI: 1.05–3.13). In Kibera, over-prescription was more common among visits with concurrent signs of respiratory infection (difficulty breathing; aOR: 3.97, 95%CI: 1.28–12.30, cough: aOR: 1.42, 95%CI: 1.06–1.90) and less common among children aged < 1 year (aOR: 0.82, 95%CI: 0.71–0.94).

**Conclusions:**

Inappropriate antibiotic prescription was common in childhood diarrhea management and efforts are needed to promote rational antibiotic use. Interventions to improve antibiotic use for diarrhea should consider the influence of malaria diagnosis on clinical decision-making and address both over-prescription, under-prescription, and inappropriate antibiotic selection.

**Electronic supplementary material:**

The online version of this article (10.1186/s12889-019-6771-8) contains supplementary material, which is available to authorized users.

## Background

Inappropriate antibiotic use, which includes prescribing unnecessary antibiotics, prescribing incorrect antibiotics, and not prescribing antibiotics when needed, is a major public health problem [1]. Overuse and misuse of antibiotics adds to the cost of treatment, risks adverse reactions and enhances the development of resistant pathogens [2]. According to a recent report on global antimicrobial resistance by the World Health Organization (WHO), antimicrobial resistance among pathogens responsible for common infections is alarmingly high [3]. In order to combat pressing public health concerns of antimicrobial resistance, a global action plan was adopted by the World Health Assembly in 2015 [4]. Increasing adherence to standard treatment guidelines has been highlighted as a core action to promote rational use of antibiotics [5, 6].

Antibiotics are frequently prescribed for common clinical syndromes, yet are not recommended for the majority of diarrheal episodes. For childhood diarrhea in resource-poor settings, WHO guidelines for the Integrated Management of Childhood Illness (IMCI) recommend empirical use of antibiotics only for dysentery, or bloody diarrhea [7], including high HIV prevalence settings [8]. For dysentery, antibiotics are intended to treat shigellosis which causes the most episodes of bloody diarrhea in children [9, 10], and ciprofloxacin is the recommended first-line agent. The recommended management of watery childhood diarrhea is oral rehydration and zinc [9] as they are most commonly caused by viral infections.

Diarrheal disease remains a major cause of morbidity and mortality among children under 5 years in Kenya [11], and *Shigella* bacteria has been frequently isolated in patients with dysentery, 66% for toddlers and 78% for children [12]. The Ministry of Health’s recommended management of diarrhea in Kenya is consistent with the WHO IMCI guidelines [13]. Although antibiotic overuse has been reported to be common in Kenya [14], little is known about specific antibiotic prescription practices for the management of childhood diarrhea in primary care facilities and their driving factors. In the current analysis, we aimed to measure the prevalence of inappropriate antibiotic prescription for childhood diarrhea case management and to determine factors associated with antibiotic over-prescription and under-prescription in one urban and one rural primary care facility in Kenya.

## Methods

### Study setting and data collection

We used data from the ongoing Population-Based Infectious Disease Surveillance (PBIDS) platform of the Kenya Medical Research Institute (KEMRI) and U.S. Centers for Disease Control and Prevention (CDC) in Kenya, described elsewhere [15]. In brief, approximately 25,000 individuals are under surveillance in each of two sites; a rural, sparsely populated, malaria-endemic setting in western Kenya (Asembo) and an urban, non-malaria endemic, densely populated, informal settlement in Nairobi, Kenya (Kibera). PBIDS participants receive free medical care for acute illness, including medications, at centrally located surveillance clinics. In Asembo all PBIDS households are within a ~ 5 km radius of the St. Elizabeth Lwak Mission Hospital, which offers predominantly outpatient services but also has a small inpatient ward. In Kibera all PBIDS households are within a ~ 1 km radius of the Tabitha clinic, which offers outpatient services only.

At the surveillance clinics, a structured questionnaire is administered by trained study staff to collect information on medical history including use of prophylactic cotrimoxazole and antiretroviral medication, and reported clinical symptoms, followed by detailed history taking and physical examinations performed by clinical officers. All data are captured in an electronic medical record system, including clinical diagnoses, any medications prescribed and if the patient illness met the case definitions of one of the four syndromes under surveillance (acute respiratory infection, acute lower respiratory infection, diarrhea, and acute febrile illness) [15]. Patients meeting the case definition for diarrhea (3 or more loose stools in the prior 24 h), are asked to provide a whole stool sample; however, stool testing results are not available immediately to guide clinical management during the clinic visit at which they are collected due to processing times. In addition, both facilities conduct on-site microscopic examination of stool for ova and parasites, and peripheral blood smear for malaria at the discretion of the clinician but not systematically for surveillance purposes.

### Inclusion, exclusion, and outcome criteria

We identified surveillance records of clinic visits of PBIDS participants aged 2–59 months presenting with diarrhea from August 21, 2009 to May 3, 2016. We then excluded records in which there was a non-diarrheal indication for antibiotics per IMCI guidelines, including those meeting criteria for possible serious bacterial infection, severe pneumonia or very severe disease, pneumonia, very severe febrile disease, and acute ear infection [7]. Although IMCI also recommends antibiotics for mastoiditis and severe complicated measles, the surveillance data did not contain the necessary variables to identify such cases based on clinical criteria; however visits with a discharge diagnosis of either of these were excluded. We also excluded records with a discharge diagnosis of meningitis, pneumonia, acute otitis media, or bacteremia, regardless of whether IMCI criteria for antibiotics were met. Since we were interested in the use of antibiotics for diarrhea based on clinical signs and symptoms, we also excluded records with a discharge diagnosis of amoebiasis or giardiasis, since microscopy results may have prompted prescription of antibiotics for those cases. Clinic visits with missing variables were excluded as we weren’t able to assess antibiotic indication per IMCI guidelines.

The remaining diarrheal cases were categorized into dysentery and non-dysentery, based on parental report and/or clinician observation of blood in the stool. We reviewed treatment data for any antibiotics prescribed during the clinical visit encounter. Antibiotic over-prescription was defined as an antibiotic prescription for non-dysentery cases and under-prescription as no antibiotic prescription for dysentery cases. Inappropriate antibiotic selection was defined as prescription of non-recommended antibiotic for dysentery cases by the guidelines [16, 17].

### Statistical analysis

Statistical analyses were performed separately for each site due to heterogeneity between the two settings. Comparison between groups were performed by Pearson chi-squared or Fisher’s exact test for categorical variables and Student’s *t*-test for continuous variables. Multivariate logistic regression was used to identify factors associated with antibiotic over-prescription and under-prescription for each site. For multivariate analyses, variables were initially selected using a cut-off *p*-value of 0.2 based on univariate analyses and final multivariate models were constructed using the least absolute shrinkage and selection operator regression [18]. Interaction terms were explored for any statistical significance and multiple stopping criteria were used to identify the best fit model. To account for clustering of antibiotic prescription tendency by clinical officers, we also adjusted for the standard errors using generalized estimating equations [19]. All statistical analyses were performed in SAS Software version 9.3 (SAS Institute, Inc., Cary, NC). All tests were two-sided and used the 5% level of significance.

## Results

**Fig. 1:**
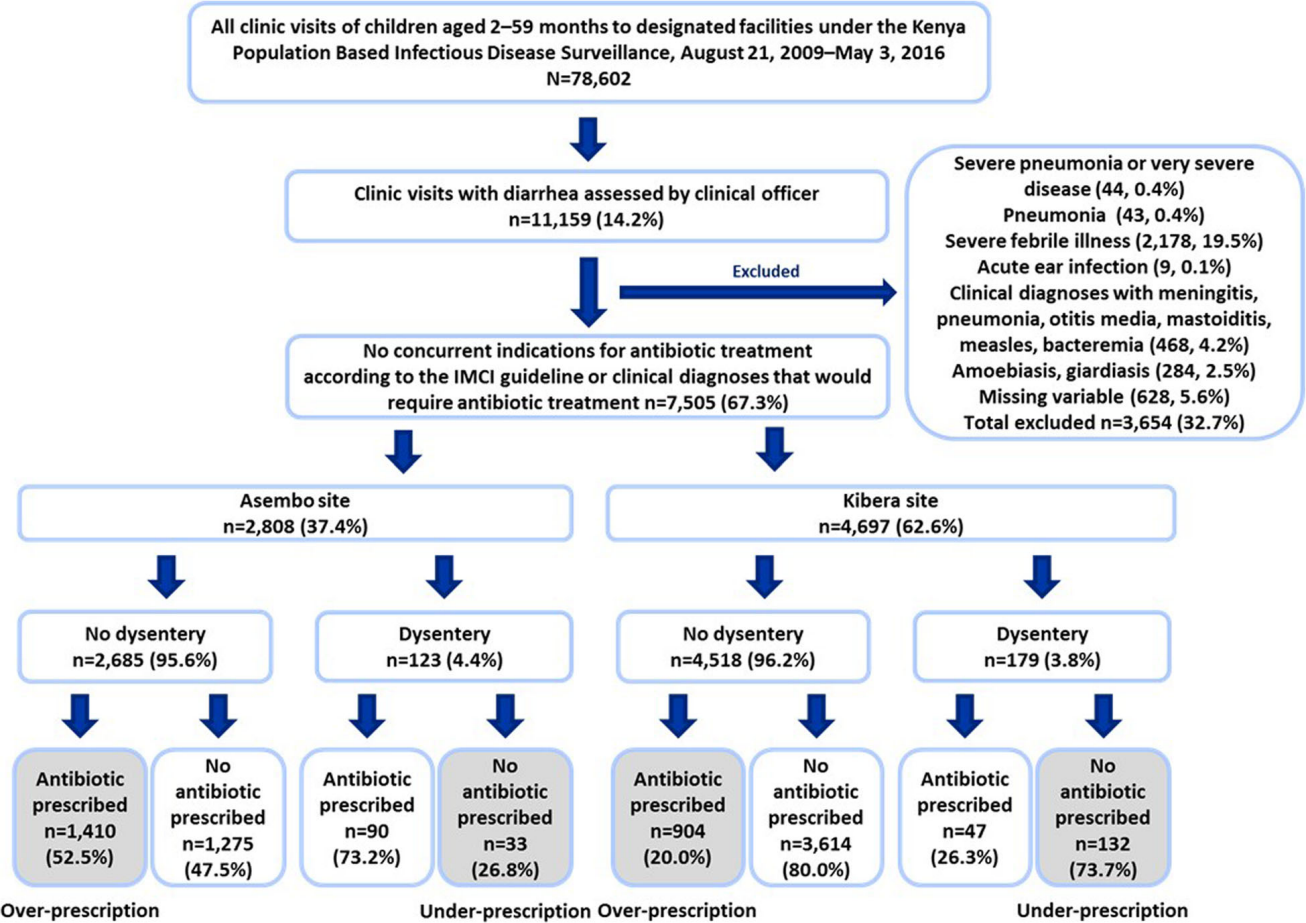
Classification of diarrheal children under five by Integrated Management of Childhood Illness guidelines in Kenya Population Based Infectious Disease Surveillance, August 21, 2009–May 3, 2016

Of 78,602 clinic visits of children aged 2–59 months in the study period, 11,159 (14.2%) were clinic visits of patients with diarrhea (Fig. 1). We excluded 3654 (32.7%) clinic visits with a non-diarrheal indication for antibiotics as defined on the Additional file 1:Table S1, and 284 (2.5%) with a diagnosis of giardiasis or amoebiasis, leaving 7505 (67.3%) visits for final analyses, including 2808 and 4697 visits for Asembo and Kibera respectively. In Asembo, 2685 (95.6%) visits were non-dysentery and antibiotic over-prescription prevalence was 52.5%. Of 123 (4.4%) dysentery visits in Asembo, antibiotic under-prescription prevalence was 26.8%. In Kibera, 4518 (96.2%) visits were non-dysentery and antibiotic over-prescription prevalence was 20.0%. Of 179 (3.8%) dysentery visits in Kibera, antibiotic under-prescription prevalence was 73.7%. During the study period, Asembo had 41 clinical officers who treated 46 diarrheal children on average (range 1–397) and Kibera had 37 clinical officers who treated 127 diarrheal children on average (range 1–1199).

Antibiotic over-prescription was more common in Asembo than Kibera (52.5% vs. 20.0%, *p* < 0.01) and factors associated with over-prescription varied between the sites (Tables 1, 2). In Asembo, antibiotic over-prescription was positively associated with a discharge diagnosis of gastroenteritis [OR: 8.23 95% CI: 3.68–18.40] but negatively associated with a diagnosis of malaria [OR: 0.37 95%CI: 0.25–0.54] and concurrent oral rehydration solution (ORS) treatment [OR: 0.49 95%CI: 0.33–0.71]. We found a significant interaction between gastroenteritis diagnosis and concurrent ORS treatment in Asembo (Additional file 1: Table S2). The ORS treatment prescription with documented gastroenteritis diagnosis was negatively associated with antibiotic over-prescription [OR: 0.35 95% CI: 0.22–0.54] while ORS treatment without documented gastroenteritis diagnosis was not associated with antibiotic over-prescription. In Kibera, antibiotic over-prescription was positively associated with prior antibiotic treatment at the surveillance facility within the last 14 days, measured or reported fever, cough, difficulty breathing, and abnormal auscultation findings of crackles or rales. Association with gastroenteritis diagnosis or ORS treatment was not found in Kibera. Age less than 1 year was negatively associated with antibiotic over-prescription [OR: 0.82 95%CI: 0.71–0.94].

**Table 1:** Multivariate analysis for factors associated with antibiotic over-prescription for diarrheal children under 5 years old without dysentery in Asembo (*N* = 2685)

Factors	Antibiotic prescribed (%)	Antibiotic not prescribed (%)	Crude odds ratio (95% CI)	Adjusted^b^ odds ratio (95% CI)
Demographics				
Age < 1	545/1410 (38.3%)	408 /1275 (32.0%)	1.32 (1.13–1.55)	
Male	745/1410 (52.8%)	707/1275 (55.5%)	1.11 (0.95–1.29)	
Recent medical history				
Clinic visit within 14 days	182/1410 (12.9%)	127/1275 (10.0%)	1.34 (1.05–1.70)	
Clinic visit within 14 days with diarrheal symptom	43/1406 (3.1%)	25/1270 (2.0%)	1.57 (0.95–2.59)	
Clinic visit within 14 days with antibiotic treatment	64/1410 (4.5%)	42/1275 (3.3%)	1.40 (0.94–2.08)	
Signs and symptoms				
Weight-for-age < −2	263/1395 (18.9%)	254/1262 (20.1%)	0.92 (0.76–1.12)	
Height-for-age < −2	369/1320 (28.0%)	324/1201 (27.0%)	1.05 (0.88–1.25)	
Weight-for-height < −2	182/1302 (14.0%)	182/1181 (15.4%)	0.89 (0.71–1.11)	
Measured or reported fever	1158/1410 (82.1%)	1112/1275 (87.2%)	0.67 (0.54–0.83)	
Cough	691/1410 (49.0%)	601/1275 (47.1%)	1.08 (0.93–1.25)	
Difficulty breathing	12/1409 (0.9%)	11/1274 (0.9%)	0.99 (0.43–2.24)	
Sore throat	12/1358 (0.9%)	4/1266 (0.3%)	2.81 (0.90–8.72)	5.03 (0.73–34.91)
Vomiting	469/1410 (33.3%)	462/1272 (36.3%)	0.87 (0.75–1.03)	0.79 (0.58–1.07)
Runny nose	477/1410 (33.8%)	416/1275 (32.6%)	1.06 (0.90–1.24)	
Sneezing	251/1409 (17.8%)	202/1275 (15.8%)	1.15 (0.94–1.41)	
Physical examination				
Restless or irritable	80/1409 (5.7%)	46/1270 (3.6%)	1.60 (1.11–2.32)	1.39 (0.81–2.40)
Sunken eyes	128/1408 (9.1%)	78/1270 (6.1%)	1.53 (1.14–2.05)	1.22 (0.80–1.84)
Drinks eagerly, thirsty	58/1407 (4.1%)	36/1268 (2.8%)	1.47 (0.96–2.25)	
Skin tenting	87/1405 (6.2%)	46/1270 (3.6%)	1.76 (1.22–2.53)	
Signs of anemia	30/1378 (2.2%)	36/1243 (2.9%)	0.75 (0.46–1.22)	
Crackles/rales	3/1408 (0.2%)	6/1272 (0.5%)	0.45 (0.11–1.81)	
Clinical diagnoses				
Gastroenteritis	1292/1410 (91.6%)	792/1275 (62.1%)	6.68 (5.36–8.32)	**8.23 (3.68–18.40)**
Malaria	322/1410 (22.8%)	660/1275 (51.8%)	0.27 (0.23–0.33)	**0.37 (0.25–0.54)**
Dehydration	123/1410 (8.7%)	66/1275 (5.2%)	1.75 (1.28–2.39)	
URTI^a^	415/1410 (29.4%)	352/1275 (27.6%)	1.09 (0.93–1.29)	
Prescription				
Concurrent ORS^a^	1130/1410 (80.1%)	830/1275 (65.1%)	2.16 (1.82–2.58)	**0.49 (0.33–0.71)**
Concurrent zinc	765/1410 (54.3%)	714/1275 (56.0%)	0.93 (0.80–1.09)	

^a^*URTI* Upper respiratory tract infection, *ORS* Oral rehydration solution

^b^Adjusted by variables shown with adjusted OR and within-clinician variation

Odd ratio in bold indicates *p*-value < 0.05

**Table 2:** Multivariate analysis for factors associated with antibiotic over-prescription for diarrheal children under 5 years old without dysentery in Kibera (*n* = 4518)

Factors	Antibiotic prescribed (%)	Antibiotic not prescribed (%)	Crude odds ratio (95% CI)	Adjusted^b^ odds ratio (95% CI)
Demographics				
Age < 1	194/904 (21.5%)	938/3614 (26.0%)	0.78 (0.65–0.93)	**0.82 (0.71–0.94)**
Male	445/904 (49.2%)	1763/3614 (48.8%)	1.02 (0.88–1.19)	
Recent medical history				
Clinic visit within 14 days	111/904 (12.3%)	456/3614 (12.6%)	0.97 (0.77–1.21)	
Clinic visit within 14 days with diarrheal symptom	40/872 (4.6%)	147/3446 (4.3%)	1.08 (0.75–1.54)	
Clinic visit within 14 days with antibiotic treatment	39/904 (4.3%)	78/3614 (2.2%)	2.04 (1.38–3.02)	**2.51 (1.72–3.69)**
Signs and symptoms				
Weight-for-age < −2	164/888 (18.5%)	625/3567 (17.5%)	1.07 (0.88–1.29)	
Height-for-age < − 2	313/806 (38.8%)	1255/3010 (41.7%)	0.89 (0.76–1.04)	
Weight-for-height < −2	58/792 (7.3%)	233/2989 (7.8%)	0.94 (0.69–1.26)	
Measured or reported fever	188/902 (20.8%)	360/3605 (9.99%)	2.37 (1.96–2.88)	**2.24 (1.56–3.23)**
Cough	505/904 (55.9%)	1667/3612 (46.2%)	1.48 (1.28–1.71)	**1.42 (1.06–1.90)**
Difficulty breathing	8/865 (0.9%)	7/3185 (0.2%)	4.23 (1.53–11.72)	**3.97 (1.28–12.30)**
Sore throat	6/901 (0.7%)	24/3599 (0.7%)	1.00 (0.41–2.45)	
Vomiting	98/904 (10.8%)	575/3611 (15.9%)	0.64 (0.51–0.81)	0.74 (0.53–1.03)
Runny nose	491/904 (54.3%)	1848/3612 (51.2%)	1.13 (0.98–1.31)	
Sneezing	97/903 (10.7%)	480/3612 (13.3%)	0.79 (0.62–0.99)	0.74 (0.40–1.38)
Physical examination				
Restless or irritable	8/903 (0.9%)	33/3585 (0.9%)	0.96 (0.44–2.09)	
Sunken eyes	11/904 (1.2%)	58/3591 (1.6%)	0.75 (0.39–1.44)	
Drinks eagerly, thirsty	11/904 (1.2%)	33/3598 (0.9%)	1.33 (0.67–2.64)	
Skin tenting	10/904 (1.1%)	45/3592 (1.3%)	0.88 (0.44–1.76)	
Signs of anemia	1/901 (0.1%)	9/3598 (0.3%)	0.44 (0.06–3.50)	
Crackles/rales	32/904 (3.5%)	41/3614 (1.1%)	3.20 (2.00–5.11)	**2.66 (1.34–5.30)**
Clinical diagnoses				
Gastroenteritis	670/904 (74.1%)	2555/3588 (71.2%)	1.16 (0.98–1.37)	1.62 (0.94–2.80)
Malaria diagnosis	15/904 (2.0%)	94/3588 (2.6%)	0.63 (0.36–1.09)	
Dehydration	5/904 (0.6%)	44/3588 (1.2%)	0.45 (0.18–1.13)	
URTI^a^	415/904 (45.9%)	1651/3588 (46.0%)	1.00 (0.86–1.15)	0.82 (0.52–1.31)
Treatment				
Concurrent ORS^a^	578/904 (63.9%)	2420/3614 (67.0%)	0.88 (0.75–1.02)	0.81 (0.54–1.22)
Concurrent zinc	335/904 (37.1%)	1327/3614 (36.7%)	1.02 (0.87–1.18)	

^a^*URTI* Upper respiratory tract infection, *ORS* Oral rehydration solution

^b^Adjusted by variables shown with adjusted OR and within-clinician variation

Odd ratio in bold indicates *p*-value < 0.05

The proportion of visits with dysentery was similar across sites (4.4% is Asembo and vs. 3.8% in Kibera, *p* = 0.22), but antibiotic under-prescription was more common in Kibera than Asembo (73.7% vs. 26.8%, *p* < 0.01) (Tables 3, 4). In Asembo, under-prescription was associated with vomiting [OR: 4.36 95%CI: 1.98–9.57] and a discharge diagnosis of malaria [OR: 1.82 95%CI: 1.05–3.13]. In Kibera, under-prescription was positively associated with age less than 1 year [OR: 4.76 95%CI: 1.46–15.5], and negatively associated with a discharge diagnosis of dysentery [OR: 0.10 95%CI: 0.03–0.34].

**Table 3:** Multivariate analysis for factors associated with antibiotic under-prescription for diarrheal children under 5 years old with dysentery in Western Kenya, Asembo (*n* = 123)

Factors	Antibiotic prescribed (%)	Antibiotic not prescribed (%)	Crude odds ratio (95% CI)	Adjusted^b^ odds ratio (95% CI)
Demographics				
Age < 1	29/90 (32.2%)	12/33 (36.4%)	1.20 (0.52–2.77)	
Male	50/90 (55.6%)	13/33 (39.4%)	0.52 (0.23–1.17)	
Recent medical history				
Clinic visit within 14 days	8/90 (8.9%)	3/33 (9.1%)	1.03 (0.26–4.12)	
Clinic visit within 14 days with diarrheal symptom	4/90 (4.4%)	1/33 (3.0%)	0.67 (0.07–6.24)	
Clinic visit within 14 days with antibiotic treatment	5/90 (5.6%)	0/33 (0%)	N/A	
Signs and symptoms				
Weight-for-age < −2	17/89 (19.1%)	9/33 (27.3%)	1.59 (0.63–4.03)	
Height-for-age < −2	22/85 (25.9%)	6/30 (20.0%)	0.72 (0.26–1.98)	
Weight-for-height < −2	14/85 (16.5%)	5/30 (16.7%)	1.02 (0.33–3.10)	
Measured or reported fever	81/90 (90.0%)	31/33 (93.9%)	1.72 (0.35–8.42)	
Cough	54/90 (60.0%)	22/33 (66.7%)	1.33 (0.58–3.08)	
Difficulty breathing	1 /90 (1.1%)	0/33 (0%)	N/A	
Sore throat	1/89 (0.9%)	0/32 (0%)	N/A	
Vomiting	23/90 (25.6%)	18/33 (54.5%)	3.50 (1.52–8.04)	**4.36 (1.98–9.57)**
Runny nose	27/90 (30.0%)	10/33 (30.3%)	1.02 (0.43–2.42)	
Sneezing	13/90 (14.4%)	6/33 (18.2%)	1.32 (0.46–3.81)	
Physical examination				
Restless or irritable	7/90 (7.8%)	2/33 (6.1%)	0.77 (0.15–3.88)	
Sunken eyes	8/90 (8.9%)	3/33 (12.1%)	1.41 (0.40–5.05)	
Drinks eagerly, thirsty	4/90 (4.4%)	1/33 (3.0%)	0.67 (0.07–6.24)	
Skin tenting	4/90 (4.4%)	0/33 (0%)	N/A	
Signs of anemia	4/88 (4.6%)	0/32 (0%)	N/A	
Crackles/rales	0/90 (0%)	0/33 (0%)	N/A	
Clinical diagnoses				
Gastroenteritis	42/90 (45.6%)	10/33 (30.3%)	0.52 (0.22–1.22)	
Malaria diagnosis	29/90 (32.2%)	18/33 (54.6%)	2.52 (1.12–5.71)	**1.82 (1.05–3.13)**
Dehydration	3/90 (3.3%)	3/33 (9.1%)	2.90 (0.56–15.13)	
URTI^a^	20/90 (22.2%)	8/33 (24.2%)	1.12 (0.44–2.86)	
Dysentery	47/90 (52.2%)	8/33 (24.2%)	0.29 (0.12–0.72)	0.30 (0.07–1.24)
Treatment				
Concurrent ORS^a^	59/90 (65.6%)	14/33 (42.4%)	0.39 (0.17–0.88)	0.34 (0.10–1.19)
Concurrent zinc	41/90 (45.6%)	15/33 (45.5%)	1.00 (0.45–2.22)	

^a^*URTI* Upper respiratory tract infection, *ORS* Oral rehydration solution

^b^Adjusted by variables shown with adjusted OR and within-clinician variation

Odd ratio in bold indicates *p*-value < 0.05

**Table 4:** Multivariate analysis for factors associated with antibiotic under-prescription for diarrheal children under 5 years old with dysentery in Nairobi, Kibera (*n* = 179)

Factors	Antibiotic prescribed (%)	Antibiotic not prescribed (%)	Crude odds ratio (95% CI)	Adjusted^b^ odds ratio (95% CI)
Demographics				
Age < 1	4/47 (8.51%)	33/132 (25.0%)	3.58 (1.20–10.72)	**4.76 (1.46–15.50)**
Male	21/47 (44.7%)	73/132 (55.3%)	1.53 (0.78–2.99)	
Recent medical history				
Clinic visit within 14 days	7/47 (14.9%)	21/132 (15.9%)	1.08 (0.43–2.74)	
Clinic visit within 14 days with diarrheal symptom	3/45 (6.7%)	8/127 (6.3%)	0.94 (0.24–3.71)	
Clinic visit within 14 days with antibiotic treatment	1/47 (2.1%)	3/132 (2.3%)	1.07 (0.11–10.51)	
Signs and symptoms				
Weight-for-age < −2	8/47 (17.0%)	21/132 (15.9%)	0.92 (0.38–2.25)	
Height-for-age < −2	23/42 (54.8%)	46/106 (43.4%)	0.63 (0.31–1.30)	
Weight-for-height < − 2	3/42 (7.1%)	7/106 (6.6%)	0.92 (0.23–3.74)	
Measured or reported fever	8/47 (17.0%)	13/132 (9.85%)	0.53 (0.21–1.38)	0.34 (0.11–1.05)
Cough	25/47 (53.2%)	55/132 (41.7%)	0.63 (0.32–1.22)	0.54 (0.25–1.17)
Difficulty breathing	1/43 (2.33%)	0/107 (0%)	N/A	
Sore throat	0/47 (0%)	0/131 (0%)	N/A	
Vomiting	5/47 (10.6%)	8/132 (6.1%)	0.54 (0.17–1.75)	
Runny nose	27/47 (57.5%)	63/132 (47.7%)	0.68 (0.35–1.32)	
Sneezing	6/47 (12.7%)	16/132 (12.1%)	0.94 (0.35–2.57)	
Physical examination				
Restless or irritable	0/47 (0%)	0/131 (0%)	N/A	
Sunken eyes	1/47 (2.1%)	0/132 (0%)	N/A	
Drinks eagerly, thirsty	2/47 (4.3%)	0/132 (0%)	N/A	
Skin tenting	1/47 (2.1%)	0/131 (0%)	N/A	
Signs of anemia	0/47 (0%)	1/131 (0.8%)	N/A	
Crackles/rales	0/47 (0%)	1/132 (0.8%)	N/A	
Clinical diagnoses				
Gastroenteritis	32/47 (68.1%)	79/131 (60.3%)	0.71 (0.35–1.44)	
Malaria diagnosis	1/47 (2.1%)	2/131 (1.5%)	0.71 (0.06–8.05)	
Dehydration	1/47 (2.1%)	1/131 (0.8%)	0.35 (0.02–5.78)	
URTI^a^	14/47 (29.8%)	40/131 (30.5%)	1.04 (0.50–2.14)	
Dysentery	13/47 (27.7%)	7/131 (5.3%)	0.15 (0.06–0.40)	**0.10 (0.03–0.34)**
Treatment				
Concurrent ORS^a^	37/47 (78.7%)	83/132 (62.9%)	0.46 (0.21–1.00)	
Concurrent zinc	15/47 (31.9%)	42/132 (31.8%)	1.00 (0.49–2.03)	

^a^URTI=Upper respiratory tract infection, ORS=Oral rehydration solution

^b^Adjusted by variables shown with adjusted OR and within-clinician variation

Odd ratio in bold indicates *p*-value < 0.05

**Fig. 2:**
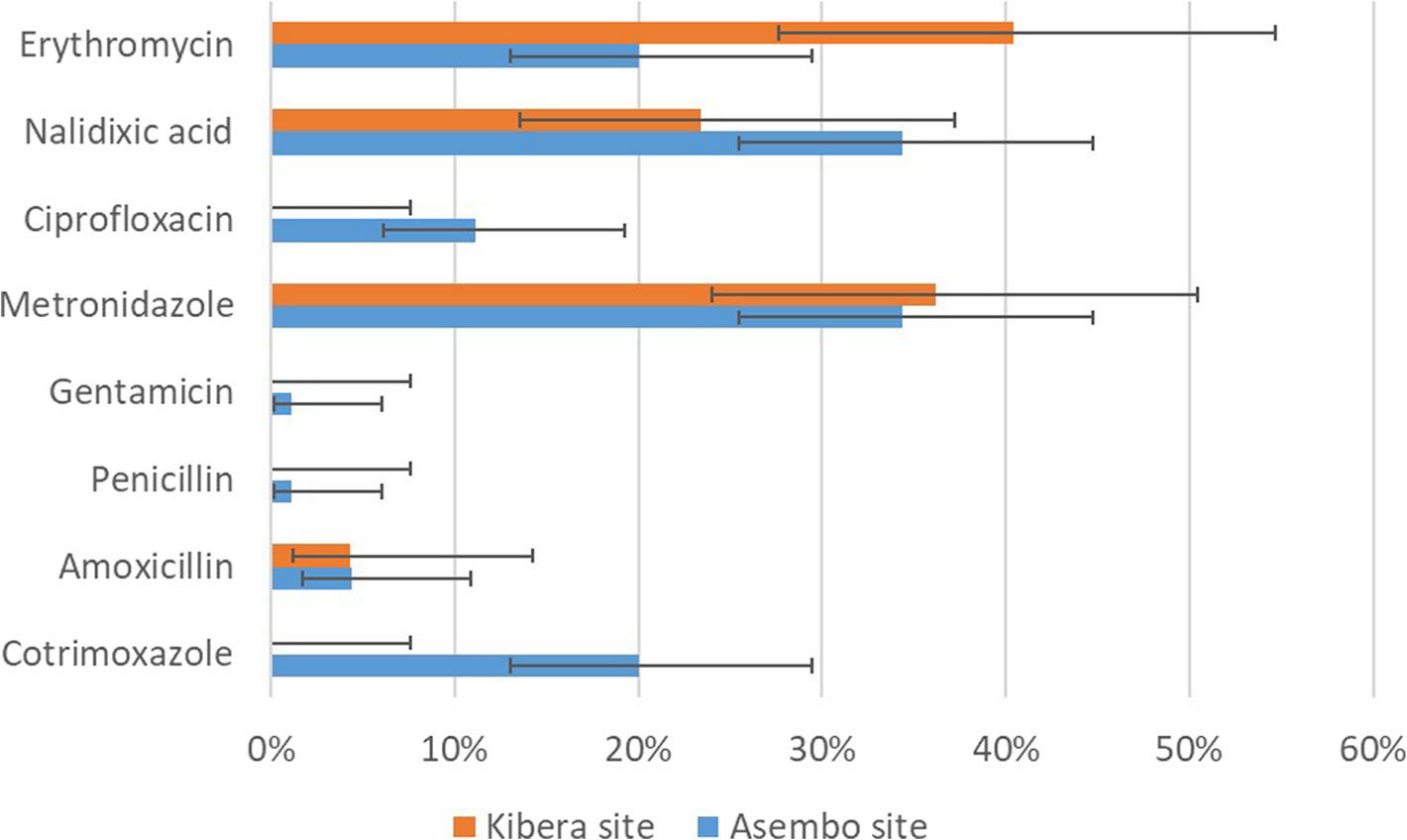
Proportion and 95% confidence interval of commonly prescribed antibiotics for diarrhea with dysentery. Antibiotics are not mutually exclusive

**Fig. 3:**
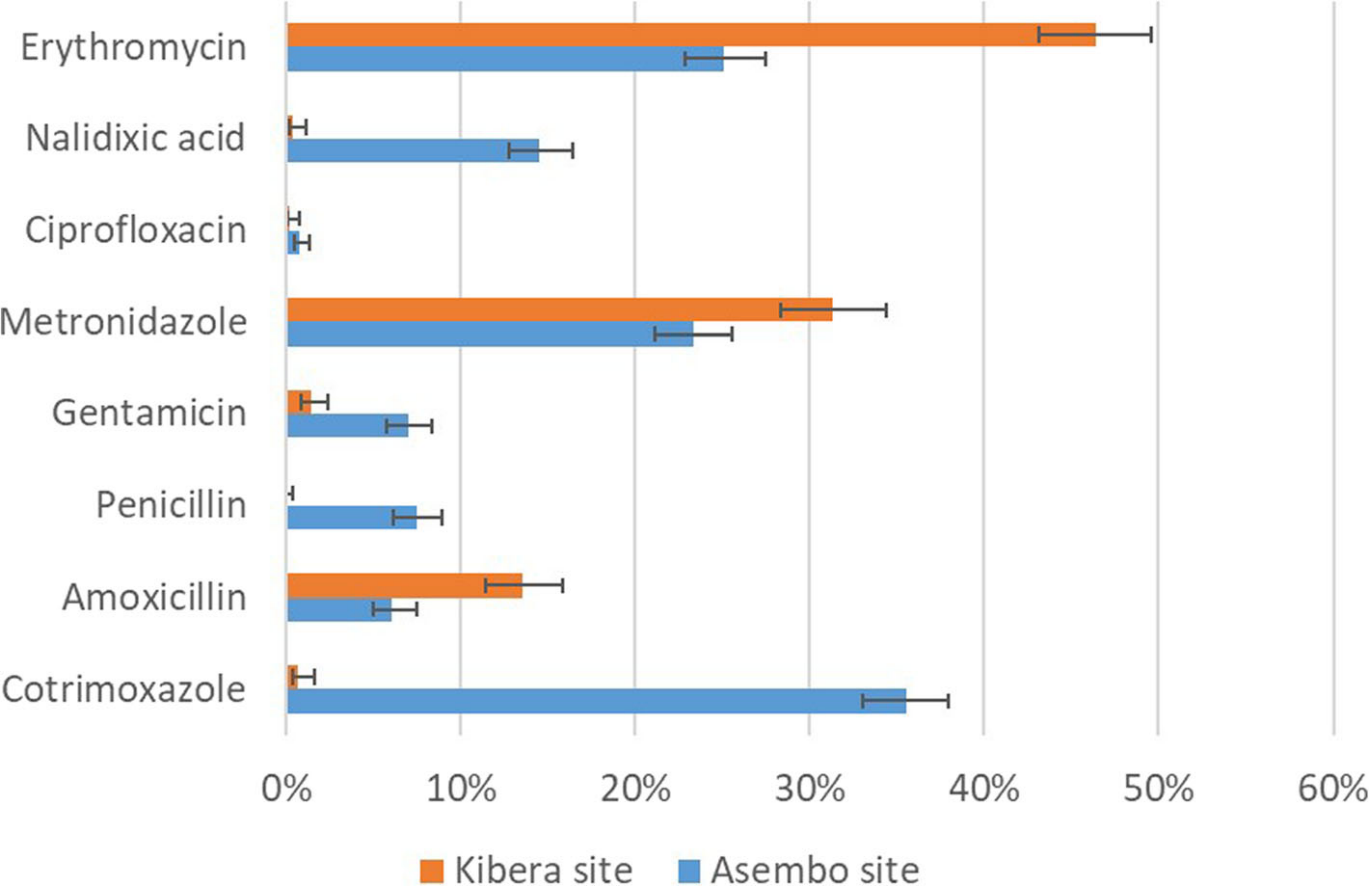
Proportion and 95% confidence interval of commonly prescribed antibiotics for diarrhea without dysentery. Antibiotics are not mutually exclusive

For dysentery visits, the most common antibiotics prescribed in both sites were erythromycin, metronidazole, and nalidixic acid (Fig. 2). Cotrimoxazole was commonly prescribed in Asembo for dysentery visits, but not used in Kibera. Ciprofloxacin, the recommended agent, was prescribed in 11% of dysentery visits in Asembo and 0% in Kibera. For diarrheal non-dysentery visits, the commonly prescribed antibiotics were erythromycin and metronidazole in Kibera and cotrimoxazole, erythromycin and metronidazole in Asembo (Fig. 3).

## Discussion

Inappropriate antibiotic prescription for childhood diarrhea management was common in both rural and urban sites in Kenya. Overall, 34.2% of diarrhea visits to health facilities had either over-prescription, under-prescription, or inappropriate choice of antibiotics. Antibiotic over-prescription was observed more frequently in the rural, malaria-endemic area while under-prescription was more predominant in the urban, densely populated, slum area. We found little overlap in the factors associated with antibiotic over-prescription and under-prescription between two study sites. Our findings indicate that despite presence of national and international guidelines, the magnitude of antibiotic over-prescription and under-prescription, and the factors that drive them, can vary greatly between facilities.

More than 95% of diarrhea clinic visits did not warrant antibiotics. Yet we found that antibiotics were inappropriately prescribed for 2 to 5 of every 10 cases of non-dysentery diarrhea. These results are consistent with other studies that have reported frequent antibiotic over-prescription for diarrhea in resource-limited outpatient settings. A cross-sectional study assessing antibiotic prescribing practice for the management of diarrhea in Tanzania reported 54.4% of children with acute watery diarrhea were prescribed with antibiotics inappropriately [20]. Similar studies in India and Thailand reported antibiotic overuse of 71 and 55.2% respectively [21, 22]. We found over-prescription to be more common in the rural site than an urban informal settlement, which is consistent with a study of antibiotic consumption in Heilongjiang Province, China [23]. Factors such as knowledge and attitude of caregiver or distance needed to travel for clinic visit might have influenced the differences between rural and urban sites. In contrast, a study conducted in Ethiopia found no difference in antibiotics overuse between rural and urban sites [24]. Despite differences between the rural and urban settings in our study, the overall frequency of inappropriate use for diarrhea is alarming, and is likely contributing to the emergence of antimicrobial resistance in Kenya [25, 26].

Factors associated with antibiotic over-prescription varied by site. In Asembo, diarrheal children were more likely to be prescribed with antibiotic when they were clinically diagnosed with gastroenteritis, suggesting that clinicians perceive acute diarrheal illness as an indication for antibiotics, despite IMCI and national guidelines recommend antibiotics only for bloody diarrhea. Furthermore, in Kibera, fever, cough, difficulty breathing and abnormal respiratory exam findings were associated with antibiotic over-prescription. This constellation of symptoms suggests that the observed antibiotic prescriptions may have been intended to treat respiratory infections. However, for this analysis we excluded clinic visits in which patients met IMCI criteria for antibiotic treatment of pneumonia or very severe pneumonia, as well as visits with a discharge diagnosis of pneumonia. Thus, any antibiotics given for respiratory infections were likely unnecessary. Antibiotic overuse for acute respiratory infections in children is common [27] and must be addressed in conjunction with overuse for diarrheal illness.

Although antibiotic over-prescription is more commonly recognized as a public health threat, we also identified frequent under-prescription when antibiotics were indicated. This is especially concerning in resource-poor settings like Kenya where the burden of infectious diseases still remains high, access to care is not always optimal, and essential drugs may not readily be available. A study evaluating the appropriateness of antibiotic use per IMCI guidelines in Papua New Guinea reported that 11% of children in outpatient settings did not receive antibiotics when they should have [28]. In our study, approximately 1 in 4 dysentery cases in Asembo and 3 in 4 cases in Kibera went untreated. *Shigella* is a common cause of bloody diarrhea and has been previously isolated from the stool of 36% of persons with dysentery in Nairobi [29], and 43.9% in rural western Kenya [30]. Moreover, stool culture unlike molecular testing, may underestimate the true burden of *Shigella* [31]. Thus, failure to treat dysentery with antibiotics represents a missed opportunity to reduce *Shigella*-related morbidity and prevent further spread of such infections in the community. In Kibera, a discharge diagnosis of dysentery was negatively associated with under-prescription, suggesting that clinician recognition of dysentery cases can improve adherence to recommended management.

In Asembo, a discharge diagnosis of malaria was inversely associated with antibiotic over-prescription but positively associated with antibiotic under-prescription. Per IMCI guidelines, a child meeting the case definition for malaria and dysentery should be treated for both. However, malaria diagnosis has been reported to increase untargeted antibiotic use [32] and affect clinician’s ability to detect other concurrent infections. A study of adherence to IMCI guidelines in Tanzania reported that health workers consistently provided recommended therapy for a single diagnosis, but rarely diagnosed or treated more than one condition [33]. A study in Mozambique found a diagnosis of malaria to be leading risk factor for failure to treat pneumonia [34]. Proper adherence to IMCI guidelines is important for avoiding unnecessary antibiotic use, but also to ensure that patients who should be treated with antibiotics receive them appropriately.

Correct choice of antibiotic agent is an important component of rational use. Although ciprofloxacin is the recommended antibiotic for dysentery in Kenya [13], the most commonly prescribed antibiotics for dysentery were metronidazole, erythromycin, and nalidixic acid. Metronidazole and erythromycin are not effective in treating shigellosis [17] and nalidixic acid, previously used as the first-line antibiotic against *Shigella*, is no longer recommended by the WHO, even in areas where *Shigella* remains susceptible [16]. Previous studies in Kenya have found *Shigella* to be susceptible to nalidixic acid [35, 36], however ciprofloxacin has a greater activity against *Enterobacteriacae* than nalidixic acid and less chance of inducing resistance in *Shigella* and other pathogens [16]. Although there are safety concerns, especially musculoskeletal adverse events, surrounding use of newer quinolones in pediatric patients [37], the WHO has deemed the benefits to outweigh the risk, but lingering concern about safety of ciprofloxacin in children may contribute to suboptimal choice of antibiotic for treating pediatric dysentery [16]. Thus adherence to the recommendation of ciprofloxacin for treatment of dysentery is important for prevention of antimicrobial resistance.

Our findings have limited generalizability as the data are from only two private health facilities with on-going disease surveillance and research activities. It is likely that inappropriate antibiotic prescribing in other facilities is more common than what we observed. We relied on data documented in the medical record; inaccurate recording of key data may have led to misclassification of antibiotic indications and factors associated with over- or under-prescription. Although the IMCI guideline recommends antibiotic treatment for diarrheal illness with a severe dehydration in the region with circulating cholera [38], we did not consider this within our study. Kenya has experienced outbreaks of cholera in 2007–2010 [39], 2014 to the present [40] and these outbreaks may have contributed to antibiotic over-prescription. However, there were very few confirmed cholera cases within the surveillance population during the study period. Moreover, due to the exclusion of records with non-diarrheal indications for antibiotics, only 1% of the included diarrheal visits were complicated by severe dehydration. Thus, cholera is unlikely to have been an important consideration in assessing use of antibiotics for childhood diarrhea. Lastly, a small number of dysentery cases may have slightly biased estimates of odds ratio for factors associated with antibiotic under-prescription.

Despite these limitations, our findings indicate that inappropriate antibiotic prescription for childhood diarrhea might be common in Kenya. Further assessment of the magnitude of inappropriate antibiotic use in a broader range of facilities and geographic locations, and to provide more insight into the factors that shape antibiotic prescription for children with diarrhea. Furthermore, in Asembo, 14% of children were given antibiotics by the caretaker before seeking care from healthcare facilities [41], suggesting future studies should also consider inappropriate antibiotic use before seeking medical care. Based on our findings, antibiotic stewardship interventions for outpatient diarrheal case management should address all aspects of inappropriate antibiotic use, including over- prescription, under-prescription and optimal choice of antibiotic agent. Strategies might include providing routine trainings on appropriate antibiotic use followed by supportive supervision and quality assurance review [42–44]. Global efforts to improve outpatient malaria diagnostic capacity should be continued with an emphasis on recognizing concurrent infections, including dysentery.

## Conclusions

Diarrhea is one of the most common reasons for children to seek medical attention in Kenya and globally; inappropriate antibiotic prescription for childhood diarrhea is an important and modifiable contributor to the growing public health threat of antimicrobial resistance.

## Additional file

Additional file 1: **Table S1.** and **Table S2.** Symptoms and signs used to define each non-diarrheal indications for antibiotics among children aged 2–59 months per Integrated Management for Childhood Illness (IMCI) guidelines are shown on **Table S1.** Data on multivariate analysis for factors associated with antibiotic over-prescription among diarrheal children under 5 years old without dysentery in Western Kenya, Asembo are shown on **Table S2.** (DOCX 17 kb)

## Abbreviations

CDC: U.S. Centers for Disease Control and Prevention
IMCI: Integrated Management of Childhood Illness
KEMRI: Kenya Medical Research Institute
PBIDS: Population-Based Infectious Disease Surveillance
WHO: World Health Organization
