# Supplementary material for: Inappropriate use of antibiotics for childhood diarrhea case management — Kenya, 2009–2016
Source: BMC Public Health. 2019 May 10;19(Suppl 3):468. doi: 10.1186/s12889-019-6771-8 (PMC6696675; doi:10.1186/s12889-019-6771-8)
Supplement: Supplementary file 1 — Table S1. and Table S2. Symptoms and signs used to define each non-diarrheal indications for antibiotics among children aged 2–59 months per Integrated Management for Childhood Illness (IMCI) guidelines are shown on Table S1. Data on multivariate analysis for factors associated with antibiotic over-prescription among diarrheal children under 5 years old without dysentery in Western Kenya, Asembo are shown on Table S2. (DOCX 17 kb) [file 12889_2019_6771_MOESM1_ESM.docx]

**Supplement Table 1. Non-diarrheal indications for antibiotics among children aged 2-59 months per Integrated Management for Childhood Illness (IMCI) guidelines***

| **Conditions** | **Symptoms and signs** |
| --- | --- |
| Severe pneumonia or very severe disease | Cough or difficult breathing with any one of following   - Any general danger sign (not able to drink/breastfeed, vomit everything, convulsion during current illness, and lethargic/unconscious) OR - Chest indrawing OR - Stridor in calm child |
| Pneumonia | Cough or difficult breathing AND  Fast breathing (40 breaths per minute or more) |
| Very severe febrile disease | Fever (by history, feels hot or temperature 37.5°C or higher) with any one of following   - Any general danger sign (not able to drink/breastfeed, vomit everything, convulsion during current illness, and lethargic/unconscious) OR - Stiff neck |
| Severe complicated measles* | Fever with any one of following   - Any general danger sign OR - Clouding of cornea OR - Deep or extensive mouth ulcers |
| Mastoiditis* | Ear problem AND  tender swelling behind the ear |
| Acute ear infection | Ear problem with any one of following   - Pus is seen draining from the ear OR - Ear pain |

*Surveillance data not sufficient to determine whether criteria for these conditions were met; visits were excluded if they had a discharge diagnosis of mastoiditis or measles

**Supplement Table 2. Multivariate analysis for factors associated with antibiotic over-prescription among diarrheal children under 5 years old without dysentery in Western Kenya, Asembo (N=2,685)**

|  | **Gastroenteritis diagnosis** | | | **No gastroenteritis diagnosis** | | | **ORs (95% CI) for gastroenteritis diagnosis within strata** |
| --- | --- | --- | --- | --- | --- | --- | --- |
|  | **Antibiotic prescribed**  **(n=1,292)** | **No antibiotic prescribed**  **(n=792)** | **ORs (95% CI) for concurrent** **ORS within strata** | **Antibiotic prescribed**  **(n=118)** | **No antibiotic prescribed**  **(n=483)** | **ORs (95% CI) for concurrent** **ORS within strata** |  |
| **No concurrent** **ORS*** | 182 (14.6%) | 52 (6.6%) | Ref | 91 (77.1%) | 393 (81.4%) | Ref | 13.36 (5.42-32.9)  *p* < 0.01 |
| **Concurrent** **ORS** | 1,103 (85.4%) | 740 (93.4%) | 0.35 (0.22-0.54)  *p* < 0.01 | 27 (22.9%) | 90 (18.6%) | 1.02 (0.87-1.19)  *p* = 0.30 | 3.42 (1.54-7.57)  *p* < 0.01 |

* ORS=Oral rehydration solution

†Shown ORs are adjusted by sore throat, vomiting, restless or irritable, sunken eyes, and malaria diagnosis
